# Higher PEEP improves outcomes in ARDS patients with clinically objective positive oxygenation response to PEEP: a systematic review and meta-analysis

**DOI:** 10.1186/s12871-018-0631-4

**Published:** 2018-11-17

**Authors:** Lanqi Guo, Jianfeng Xie, Yingzi Huang, Chun Pan, Yi Yang, Haibo Qiu, Ling Liu

**Affiliations:** Department of Critical Care Medicine, Zhongda Hospital, School of Medicine, Southeast University, Nanjing, Jiangsu China

**Keywords:** ARDS, PEEP, Mortality, Barotrauma, Meta

## Abstract

**Background:**

Mortality in patients with acute respiratory distress syndrome (ARDS) remains high. These patients require mechanical ventilation strategies that include high positive end-expiratory pressure (PEEP). It remains controversial whether high PEEP can improve outcomes for ARDS patients, especially patients who show improvement in oxygenation in response to PEEP. In this meta-analysis, we aimed to evaluate the effects of high PEEP on ARDS patients.

**Methods:**

We electronically searched randomized controlled trials (RCTs) reported in the MEDLINE, CENTRAL, EMBASE, CINAHL and Web of Science databases from January 1990 to December 2017. Meta-analyses of the effects of PEEP on survival in adults with ARDS were conducted using the methods recommended by the Cochrane Collaboration.

**Results:**

A total of 3612 patients from nine randomized controlled trials (RCTs) were included. There were 1794 and 1818 patients in the high and low PEEP groups, respectively. Hospital mortality showed no significant difference between the high and low PEEP groups (RR = 0.92; 95% CI, 0.79 to 1.07; *P* = 0.26). Similar results were found for 28-d mortality (RR = 0.88; 95% CI, 0.72 to 1.07; *P* = 0.19) and ICU mortality (RR = 0.83; 95% CI, 0.65 to 1.07; *P* = 0.15). The risk of clinically objectified barotrauma was not significantly different between the high and low PEEP groups (RR = 1.24; 95% CI, 0.74 to 2.09, *P* = 0.41). In the subgroup of ARDS patients who responded to increased PEEP by improved oxygenation (from 6 RCTs), high PEEP significantly reduced hospital mortality (RR = 0.83; 95% CI 0.69 to 0.98; *P* = 0.03), ICU mortality (RR = 0.74; 95% CI, 0.56 to 0.98; *P* = 0.04),but the 28-d mortality was not decreased(RR = 0.83; 95% CI, 0.67 to 1.01; *P* = 0.07). For ARDS patients in the low PEEP group who received a PEEP level lower than 10 cmH_2_O (from 6 RCTs), ICU mortality was lower in the high PEEP group than the low PEEP group (RR = 0.65; 95% CI, 0.45 to 0.94; *P* = 0.02).

**Conclusions:**

For ARDS patients who responded to increased PEEP by improved oxygenation, high PEEP could reduce hospital mortality, ICU mortality and 28-d mortality. High PEEP does not increase the risk of clinically objectified barotrauma.

**Electronic supplementary material:**

The online version of this article (10.1186/s12871-018-0631-4) contains supplementary material, which is available to authorized users.

## Background

Acute respiratory distress syndrome (ARDS) is a common clinical syndrome that has significant morbidity and mortality [1]. In patients with acute respiratory distress syndrome, mechanical ventilation with a lower tidal volume could decrease mortality [2]. Numerous approaches had been adopted to improve lung-protective ventilation strategies, but the mortality of ARDS remains high. Three large RCTs were performed to assess if higher PEEP could improve outcomes in patients with ARDS [3–5]. All of the trials failed to find that higher PEEP could improve the survival of ARDS patients. However, higher PEEP showed benefits in severe ARDS patients in subgroup analysis.

High PEEP can improve the PaO_2_/FiO_2_ compared with low PEEP. PEEP is an easily implemented intervention that is primarily used to prevent atelectasis and to correct hypoxemia caused by alveolar hypoventilation. Borges et al. showed that recruitment manoeuvres with PEEP and subsequent maintenance of a high level of PEEP reversed the collapse of alveoli and improved oxygenation [6]. Putensen’s study showed better outcomes with routine use of low tidal volume but not high PEEP ventilation in unselected patients with ARDS [7]. Recently, the Alveolar Recruitment for ARDS Trial found that in patients with moderate to severe ARDS, a strategy of lung recruitment and titrated PEEP compared with low PEEP decreased 28-day all-cause mortality [8]. A meta-analysis showed that higher PEEP could improve survival in ARDS patients with a PaO_2_/FiO_2_ less than 200 mmHg [9]. These findings indicated that higher PEEP needed to be used in patients who potentially benefit from it.

PEEP is a double-edged sword when it is used in ARDS patients. PEEP could open the collapsed alveoli in the dependent lung and may induce hyperinflation in the nondependent lung. Therefore, higher PEEP could improve outcomes based on lung recruitablity in ARDS patients [2]. According to the findings from Goligher’s study [10], patients who responded to increased PEEP by improved oxygenation, defined as positive oxygenation response to PEEP, might benefit more from higher PEEP. Thus, we speculated that PEEP could have different effects on clinical outcomes according to the nature of the clinical response to PEEP itself. Therefore, we conducted this meta-analysis to establish whether higher PEEP could improve survival among specific subgroups of ARDS patients who show improvement in oxygenation as a response to increased PEEP.

## Methods

### Eligibility criteria and information sources

We electronically searched randomized controlled trials (RCTs) published in the MEDLINE, EMBASE, CINAHL and Web of Science databases from January 1990 to December 2017. Additional files 1, 2, 3, 4, 5, 6, 7, 8, and 9 and supplementary appendices of the relevant articles were also reviewed.

### Search and study selection

To avoid bias, two investigators assessed the appropriateness of retrieved studies by considering the titles, abstracts and citations independently. The following keywords were used: “ALI” or “acute lung injury”, or “ARDS”, or “acute respiratory distress syndrome”, or “ARF”, or “acute respiratory failure”, and “PEEP”, or “positive end-expiratory pressure”, mortality, ICU mortality and barotrauma. The reviewers evaluated the studies based on the inclusion and exclusion criteria, and they worked out any differences by consensus. The study subjects were limited to “human”. Because a meta-analysis has as its research material previously published data, this research required no ethical approval or patient consent. We only included randomized controlled clinical trials that compared high PEEP with low PEEP in acute respiratory failure, acute lung injury or acute respiratory distress syndrome, whether to ventilate with low tidal volume, that provided the numbers of patients with high PEEP and low PEEP, and that reported patient mortality. We excluded retrospective studies and studies in which previously published data were re-analysed. We also excluded studies involving children and infants.

### Data items

Clinically objectified barotrauma was defined as the presence of pneumothorax on chest radiograph or chest tube insertion for any new pneumothorax, pneumomediastinum, subcutaneous emphysema, or pneumatocele after random assignment. The hospital mortality, ICU mortality and 28-day mortality and the incidence of clinically objectified barotrauma were extracted.

### Quality assessment

**Fig. 1:**
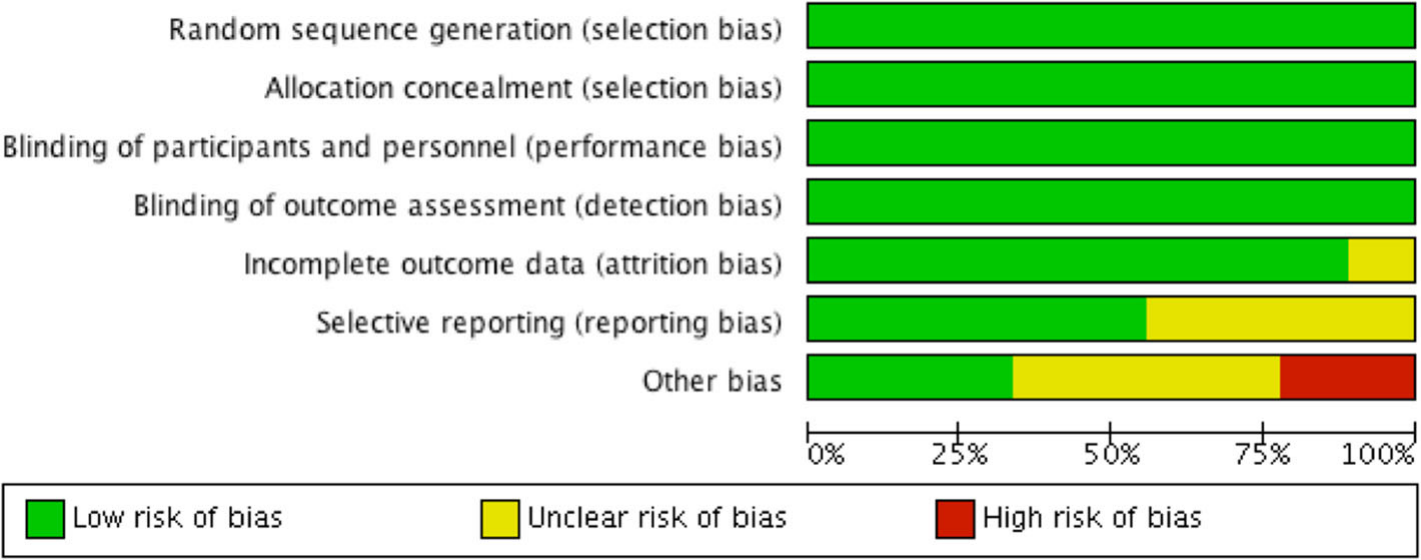
Cochrane risk of bias assessment for mortality outcome

Two reviewers independently assessed the quality of RCTs, extracted data, and cross-checked the results. Any disagreements were resolved by a third party. The quality evaluation criteria were assessed by the 5-point scale Jadad scale [5]. The total score was 1–5 (1–2: low-quality research; 3–5: high-quality research). This instrument grades (a) the use of randomization, (b) the use of blinding, (c) the handling of withdrawals and dropouts, (d) the comparability of baselines between groups, and (e) the similarity to other treatment interventions and the accuracy of intervention. The other method to assess the quality of RCTs was assessing the randomization method according to the criteria from the Cochrane Collaboration (Fig. 1).

### Sub-group analysis

According to Goligher’s study [10], we defined positive oxygenation response to PEEP as ΔPaO_2_/FiO_2_ > 0 mmHg when compared mean PaO_2_/FiO_2_ at baseline (lower PEEP) and after PEEP increased to a higher PEEP level in the high PEEP group. However, in the low PEEP group, oxygenation response to PEEP was unavailable because the baseline PEEP did not increase to a high level during the study period. We analysed the sub-groups according to whether patients in the high PEEP group had positive oxygenation response to PEEP to determine whether high PEEP could improve outcomes in patients who respond to increased PEEP by improved oxygenation. In addition, the PEEP value in the low PEEP group and the severity of ARDS also affected whether high PEEP reduced mortality. Therefore, we also performed sub-group analysis to determine whether PEEP greater than 10 cmH_2_O in the low PEEP group or baseline PaO_2_/FiO_2_ equal to or less than 150 mmHg could have some effect on mortality between the high and low PEEP groups.

### Summary measures

The meta-analysis was performed according to the Cochrane Collaboration guidelines. All statistical analyses were performed with Review Manager, version 5.3 (the Nordic Cochrane Centre, Copenhagen, Denmark), the Cochrane Collaboration’s software for preparing and maintaining Cochrane systematic reviews. The pooled-effects estimates for binary variables are expressed as risk ratios with 95% CIs, whereas continuous variables are expressed as weighted mean differences with 95% CIs.

### Synthesis of results

We tested the difference in the estimated value of the treatment effect between the experimental and control groups with a 2-sided z-test. Statistical significance was defined as *P* < 0.05. We predefined heterogeneity as low, moderate, and high with *I*^*2*^ statistics greater than 25, 50, and 75%, respectively [11]. A meta-analysis with a random-effects model was applied with *I*^*2*^ statistics greater than 50%. For other *I*^*2*^ values, a fixed-effects model was selected. Funnel plot were used to analyse publication bias.

## Results

### Study selection

**Fig. 2:**
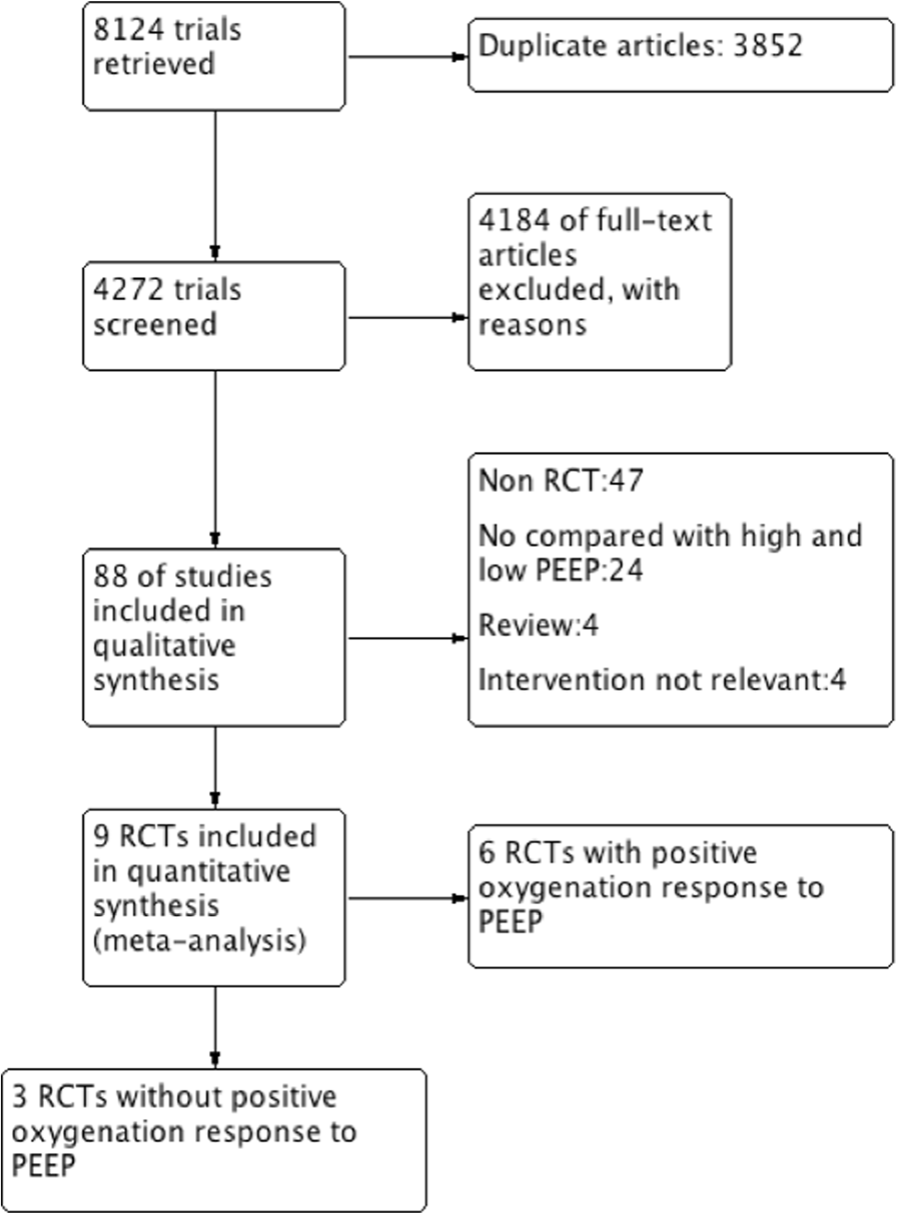
Study flow diagram

Our initial electronic and manual search identified 8124 studies. Ultimately, nine RCTs fulfilled the inclusion criteria and were included in the cumulative meta-analysis (Fig. 2). The characteristics of the included studies are shown in Table 1. A total of 3612 patients, including 1794 patients in the high PEEP group and 1818 patients in the low PEEP group, were included in our meta-analysis.

**Table 1:** Characteristics of the included patients

Trial	Jadad score	Inclusion criteria	method of PEEP selection	Number of Patients (HP/LP)	Age (HP/LP)	PEEP (cmH_2_O) (HP/LP)	Gender (HP vs. LP) [male/female]	PaO_2_/FIO_2_ (mmHg) (HP/LP)
Amato/1998 [12]	3	Lung injury score ≥ 2.5	HP^a^:LIP^c^ + 2 cmH_2_O LP^b^:FiO_2_-PEEP	29/ 24	36/33	13.2 ± 0.4/9.3 ± 0.5	–	112/134
Ranieri/1999 [13]	3	PaO_2_:FIO_2_ ≤ 200 mmHg	HP^a^:LIP^c^ + 2 cm H2O LP^b^:FiO_2_-PEEP	18/19	51/49	14.8 ± 2.7/6.5 ± 1.7	7/11 vs. 10/19	149/142
Brower/2004 [3]	4	PaO_2_:FIO_2_ ≤ 300 mmHg	HP^a^:FiO_2_-PEEP (ARDSnet) LP^b^:FiO_2_-PEEP (PEEP more than 12 cmH_2_O)	276/273	54/49	14.7 ± 3.5/8.9 ± 3.5	145/128 vs.157/119	151/165
Villar/2006 [14]	3	PaO_2_:FIO_2_ ≤ 200 mmHg	HP^a^:LIP^c^ + 2 cmH_2_O LP^b^:FiO_2_-PEEP	50/45	48/52	14.1 ± 2.8/9.0 ± 2.7	23/27 vs.27/18	111/109
Meade/2008 [5]	4	PaO_2_:FIO_2_ ≤ 250 mmHg	FiO_2_-PEEP	475/508	54.5/56.9	15.6 ± 3.9/10.1 ± 3.0	282/193 vs. 307/201	144.8/144.6
Mercat/2008 [15]	4	PaO_2_:FIO_2_ ≤ 300 mmHg	HP^a^:Pplat LP^b^: FiO_2_-PEEP	385/382	60/60	14.6 ± 3.2/7.1 ± 1.8	260/125 vs. 256/126	144/143
Talmor/2008 [16]	3	PaO_2_:FIO_2_ ≤ 200 mmHg	HP^a^:Transpulmonary pressure LP^b^:FiO_2_-PEEP	30/31	54.5/51.2	17 ± 6.0/10 ± 4.0	19/11 vs. 17/14	91/107
Huh/2009 [17]	3	PaO_2_:FIO_2_ ≤ 200 mmHg	HP^a^:saturation decrease more than 2% and drop of static compliance LP^b^:FiO_2_-PEEP	30/27	55/62		18/12 vs. 17/10	115/110
ART/2017 [8]	3	PaO_2_:FIO_2_ ≤ 200 mmHg	HP^a^: Maximum alveolar recruitment by compliance + 2 cmH_2_O LP^b^: FiO_2_-PEEP	501/509	51.3/56	16.8 ± 3.8/13.0 ± 0.3	313/188 vs. 318/191	119.5/117.2

^a^*HP* High PEEP group, ^b^*LP* Low PEEP group, ^c^*LIP* lower inflection point

### Effect of high PEEP on hospital mortality in ARDS patients

**Fig. 3:**
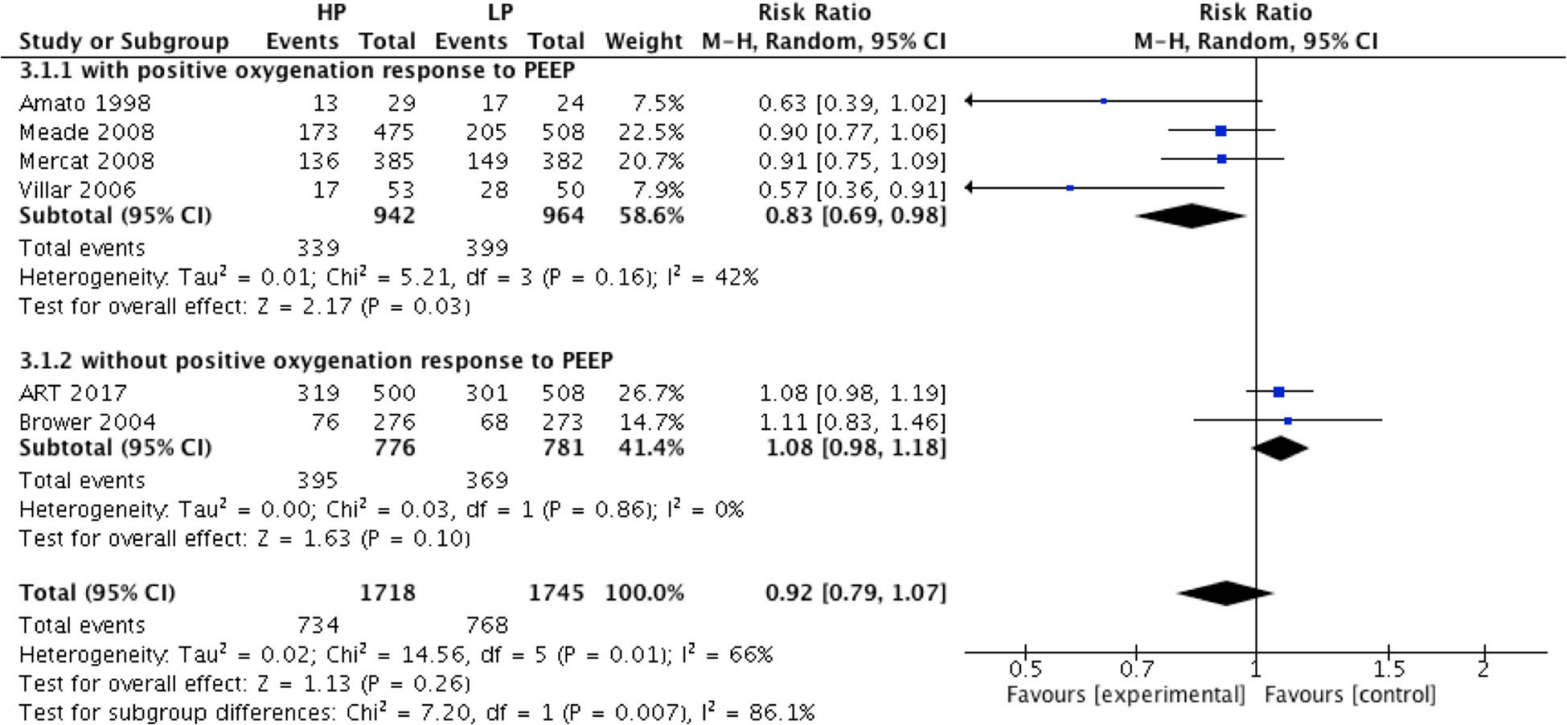
Effect of high PEEP on hospital mortality in ARDS patients with or without positive oxygenation response to PEEP

There was no difference in hospital mortality between the high PEEP and low PEEP groups (RR = 0.92; 95% CI, 0.79 to 1.07; *P* = 0.26) (Fig. 3). In ARDS with positive oxygenation response to PEEP (from 4 RCTs), hospital mortality was lower in the high PEEP group than the low PEEP group (RR = 0.83; 95% CI, 0.69 to 0.98; *P* = 0.03) (Fig. 3). However, hospital mortality was comparable between groups in patients without positive oxygenation response to PEEP (RR = 1.08; 95% CI, 0.98 to 1.18; *P* = 0.1) (Fig. 3).

**Fig. 4:**
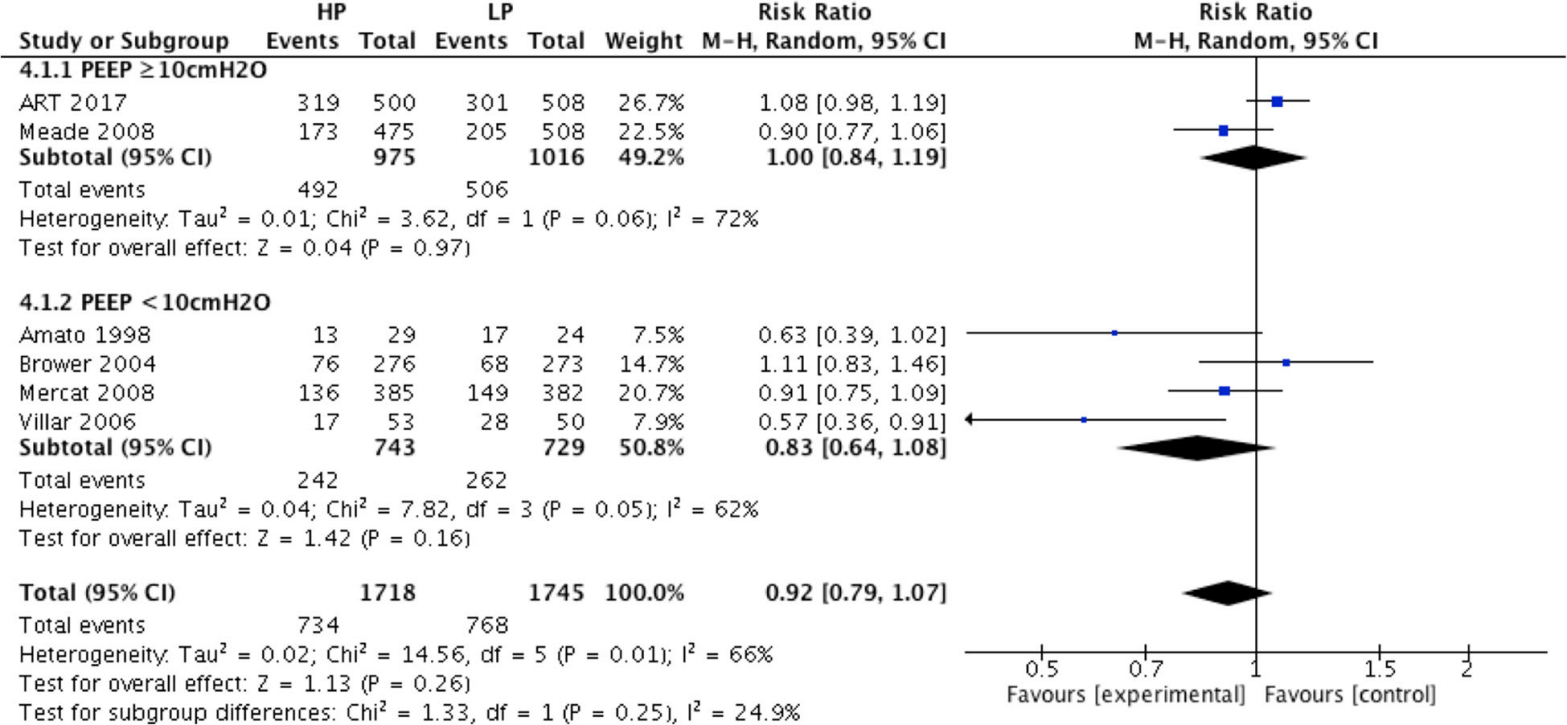
Effect of high PEEP on hospital mortality while the PEEP level of patients in low PEEP group was different

**Fig. 5:**
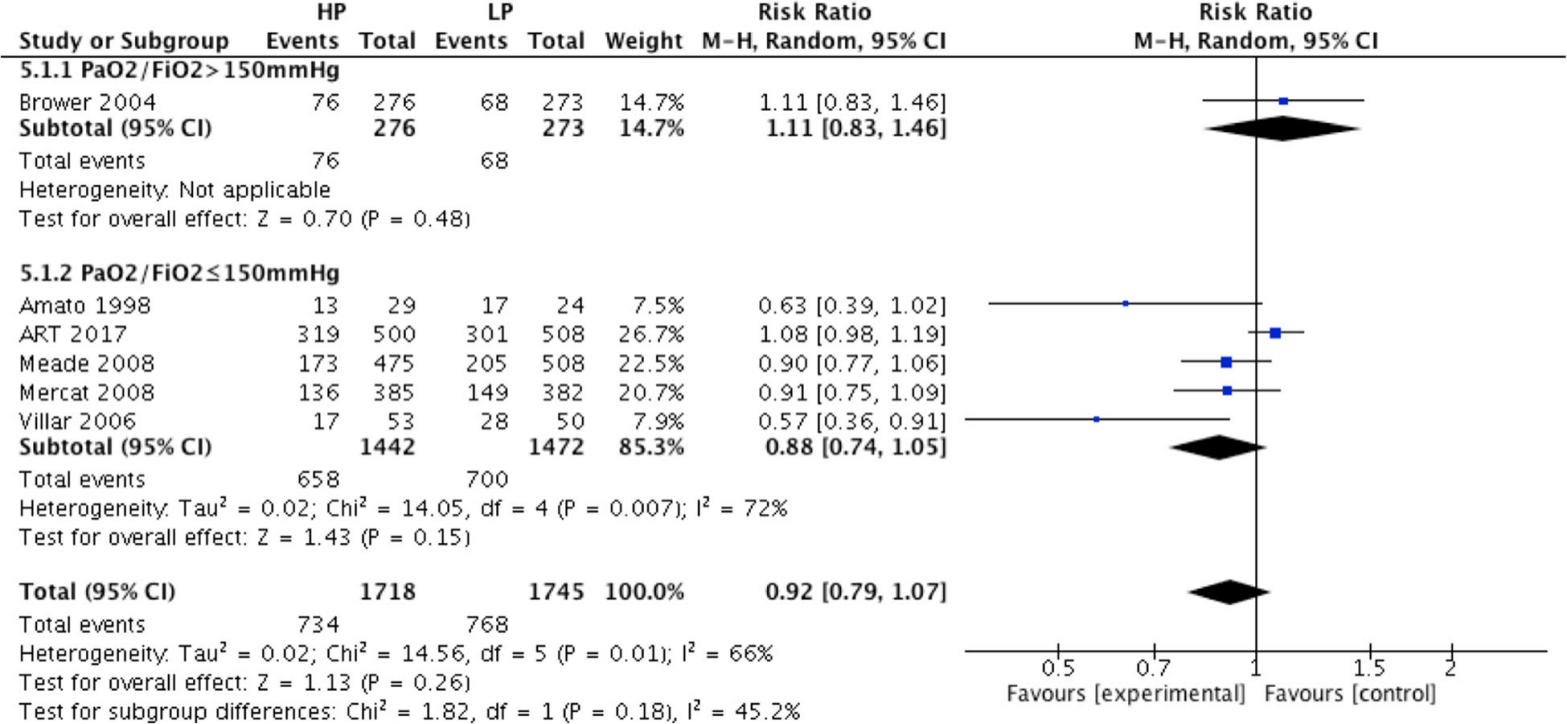
Effect of high PEEP on hospital mortality of moderate and severe ARDS patients between high and low PEEP groups

For ARDS patients in the low PEEP group who received a PEEP level lower than 10 cmH_2_O (from 4 RCTs), hospital mortality was no different in the high and low PEEP groups (RR = 0.83; 95% CI, 0.64 to 1.08; *P* = 0.16) (Fig. 4). There was no significant difference in hospital mortality between high and low PEEP groups (RR = 1.0; 95% CI, 0.84 to 1.19; *P* = 0.97) for ARDS patients in the low PEEP group who received a PEEP level equal or higher than 10 cmH_2_O (Fig. 4). For ARDS patients with baseline PaO_2_/FiO_2_ ≤ 150 mmHg (from 5 RCTs), there was no significant difference in hospital mortality between high and low PEEP groups (RR = 0.88; 95% CI, 0.74 to 1.05; *P* = 0.15) (Fig. 5).

### Effect of high PEEP on 28-day mortality in ARDS patients

**Fig. 6:**
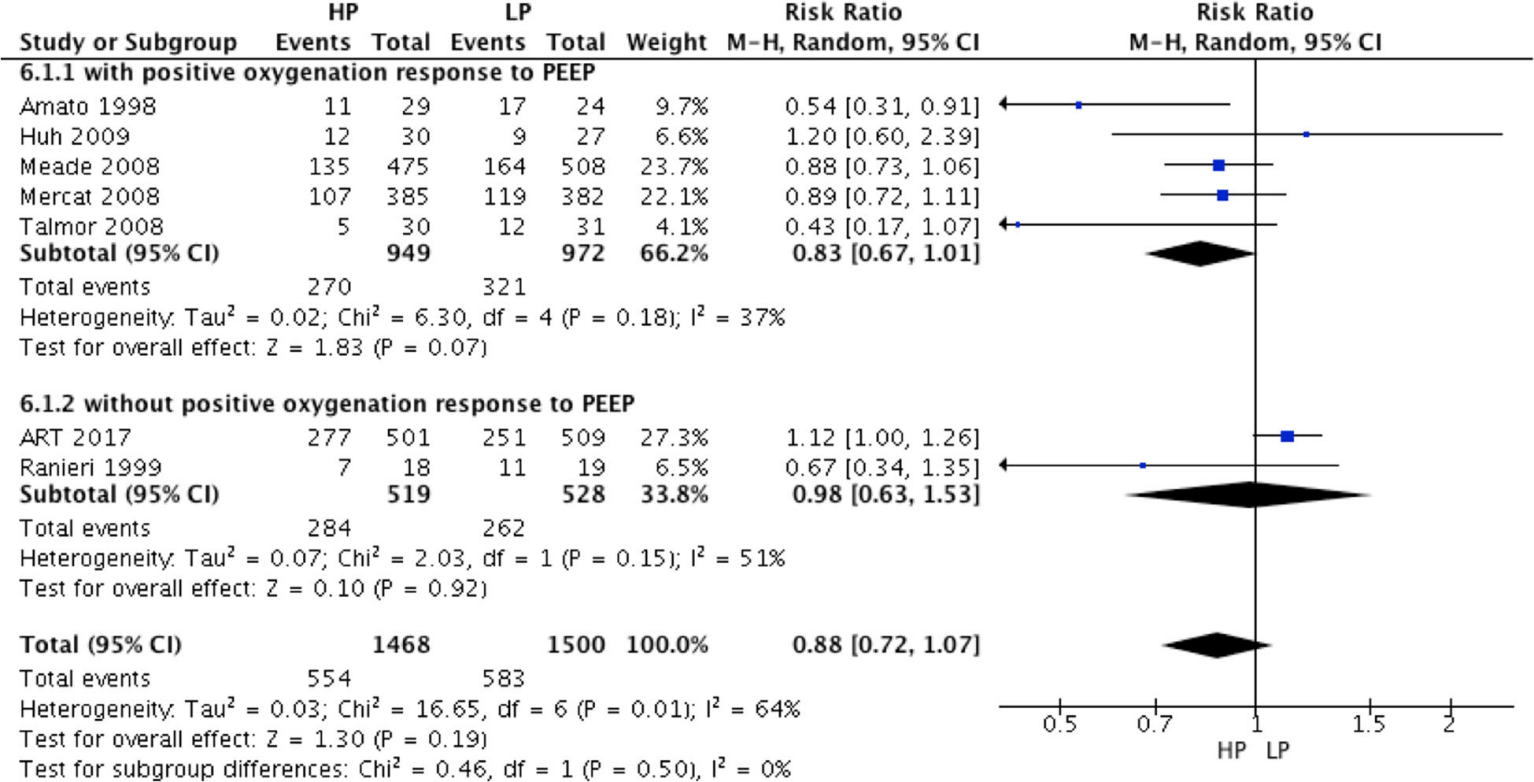
Effect of high PEEP on 28-day mortality in ARDS patients

Twenty-eight-day mortality was reported in seven RCTs and was similar in the high and low PEEP groups (RR = 0.88; 95% CI,0.72 to 1.07; *P* = 0.19) (Fig. 6). In ARDS patients with positive oxygenation response to PEEP (5 RCTs), 28-day mortality was lower in the high PEEP group than the low PEEP group (RR = 0.83; 95% CI, 0.67 to 1.01; *P* = 0.07) (Fig. 6).

**Fig. 7:**
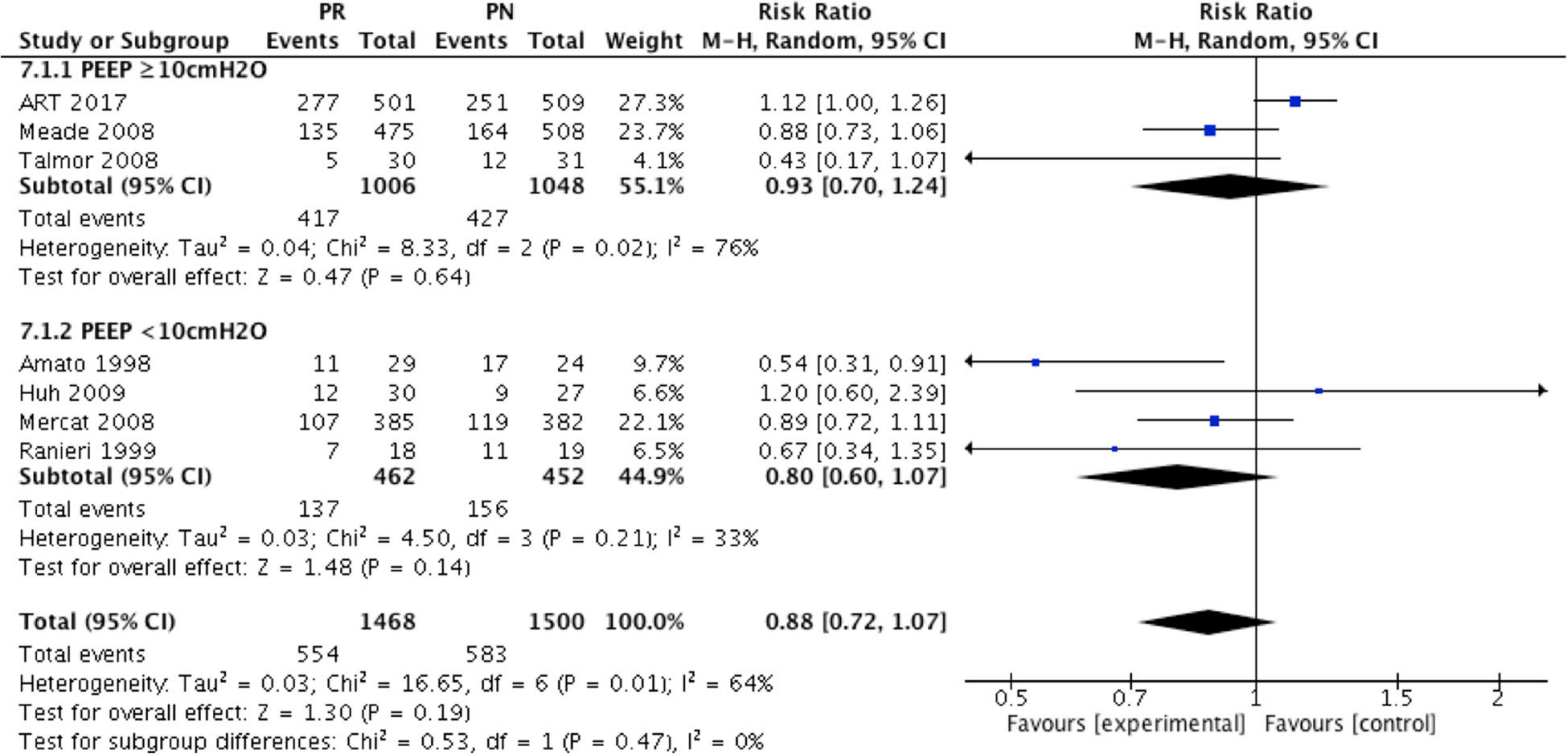
Effect of high PEEP on 28-day mortality while the PEEP level of patients in low PEEP group was different

There was no difference in 28-day mortality between the high and low PEEP groups, regardless of the PEEP level used in the low PEEP group (For PEEP less than 10 cmH_2_O, RR = 0.80; 95% CI, 0.60 to 1.07; *P* = 0.14) (For PEEP equal and more than10 cmH_2_O, RR = 0.93; 95% CI, 0.70 to 1.24; *P* = 0.64) (Fig. 7).

### Effect of high PEEP on ICU mortality in ARDS patients

**Fig. 8:**
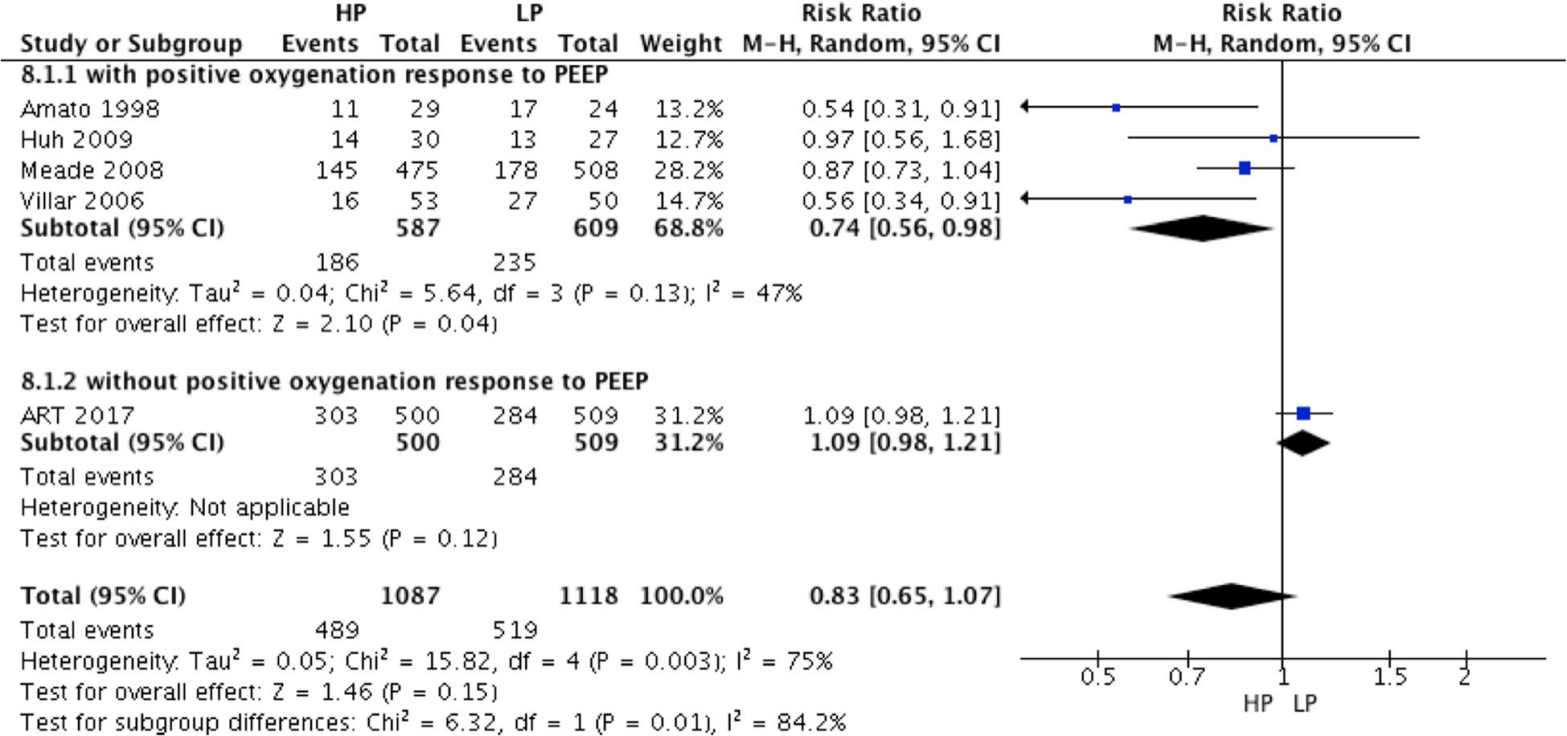
Effect of high PEEP on ICU mortality in ARDS patients

ICU mortality was reported in five RCTs. A high PEEP strategy did not decrease the ICU mortality compared to the low PEEP strategy (RR = 0.83; 95% CI, 0.65 to 1.07; *P* = 0.15) (Fig. 8). In ARDS with positive oxygenation response to PEEP (4 RCTs), ICU mortality was lower in the high PEEP group than the low PEEP group (RR = 0.74; 95% CI, 0.56 to 0.98; *P* = 0.04) (Fig. 8). However, ICU mortality was not different between the high and low PEEP groups among ARDS patients without a positive oxygenation response to PEEP.

**Fig. 9:**
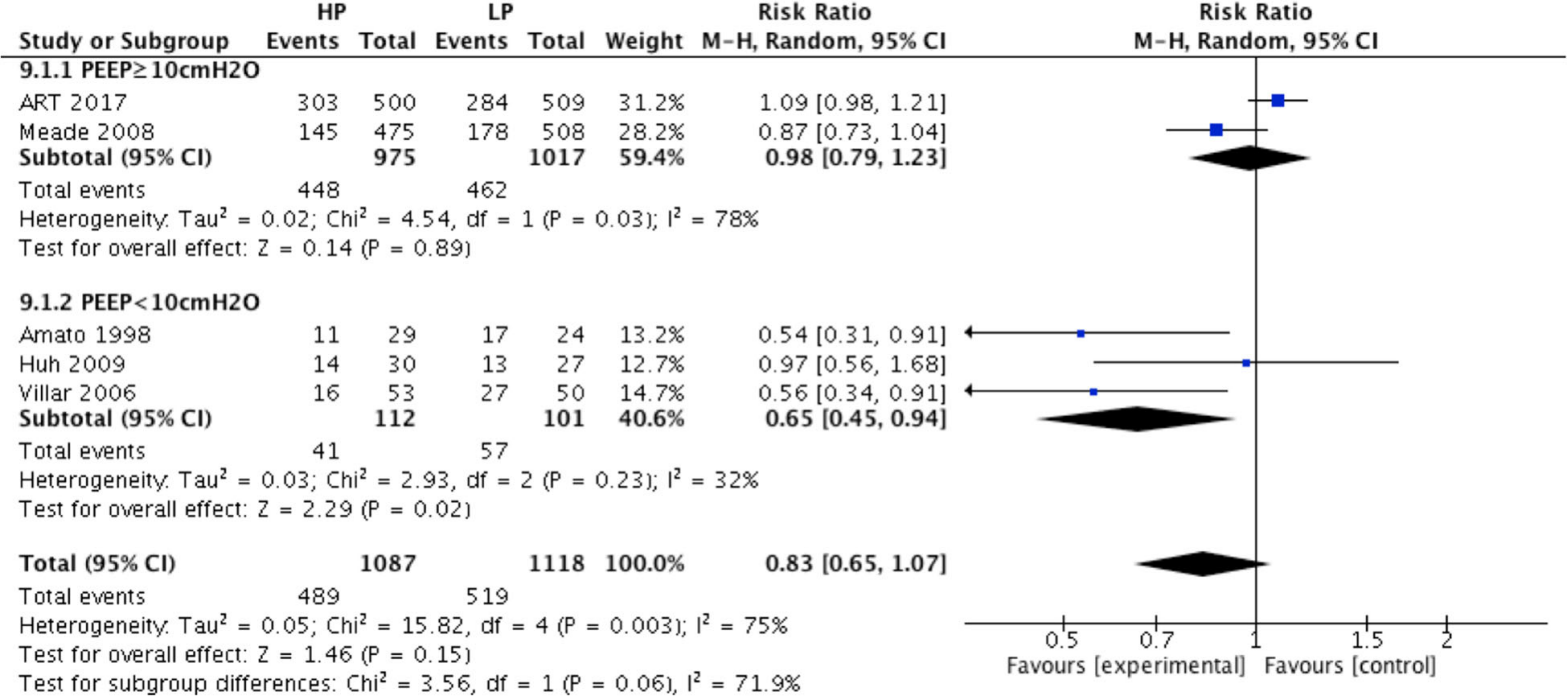
Effect of high PEEP on ICU mortality while the PEEP level of patients in low PEEP group was different

For ARDS patients in the low PEEP group who received a PEEP level lower than 10 cmH_2_O (from 3 RCTs), ICU mortality was lower in the high PEEP group than the low PEEP group (RR = 0.65; 95% CI, 0.45 to 0.94; *P* = 0.02) (Fig. 9). In contrast, there was no significant difference in ICU mortality between the high and low PEEP groups if the PEEP level was equal and more than 10 cmH_2_O in the low PEEP group (RR = 0.98; 95% CI, 0.79 to 1.23; *P* = 0.89) (Fig. 9).

### Effect of high PEEP on clinically objectified barotrauma in ARDS patients

**Fig. 10:**
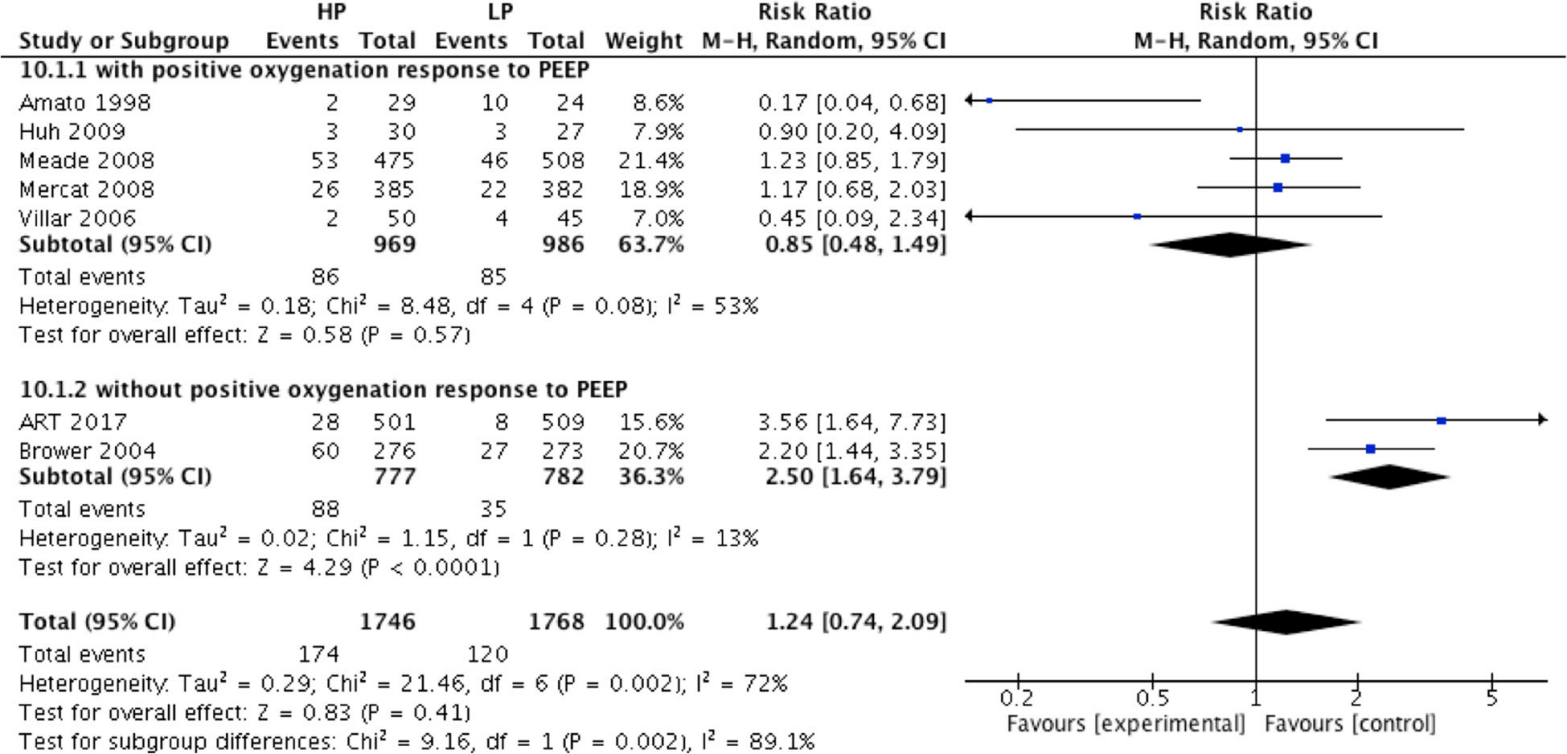
Effect of high PEEP on clinically objectified barotrauma in ARDS patients

Seven RCTs reported data on clinically objectified barotrauma, including 1746 patients in the high PEEP group and 1768 patients in the low PEEP group. A meta-analysis with a random-effects model showed that the incidence of clinically objectified barotrauma in the high and low PEEP groups were 9.97% (174/1746) and 6.8% (120/1768), respectively. High PEEP did not increase the incidence of clinically objectified barotrauma (RR = 1.24; 95% CI, 0.74 to 2.09, *P* = 0.41) (Fig. 10). However, in ARDS patients without positive oxygenation response to PEEP, high PEEP increased the incidence of clinically objectified barotrauma (RR = 2.50; 95% CI, 1.64 to 3.79, *P* < 0.0001) (Fig. 10).

## Discussion

Our meta-analysis showed that high PEEP did not improve outcomes in patients with ARDS. However, subgroup analysis showed that it may decrease hospital mortality as well as ICU and 28-d mortality in ARDS patients with clinically positive oxygenation response to PEEP. In addition, in ARDS patients without positive oxygenation response to PEEP, high PEEP increased the incidence of clinically objectified barotrauma. The results were different from Goligher’s study [18], which found that recruitment manoeuvres in combination with higher PEEP could reduce mortality in ARDS patients.

Evidence from previous studies showed that high PEEP does not decrease mortality in patients with ARDS [7, 8, 19, 20], which is in line with our results. However, several issues must be considered when interpreting this result. First, there was a large variation in the PEEP level between the different studies. For example, Huh et al. [17] set a high PEEP of 10 cmH_2_O, while the high PEEP was 16.3 cmH_2_O in Amato’s study [12]. Similarly, the PEEP level varied from 6.5 to 13 cmH_2_O in the low PEEP groups among the included studies. Therefore, the different PEEP levels may have led to the different results. Subgroup results showed that the PEEP level in the low PEEP group greatly impacted the effect of PEEP on ICU mortality. Second, patient characteristics also impacted the effect of PEEP. Among these studies, the severity of ARDS in patients varied widely. For example, baseline PaO_2_/FiO_2_ ranged from 110.8 ± 6.3 to 165 ± 77 mmHg. Oxygenation response to PEEP was also significantly associated with the severity of ARDS. The potential lung with response to PEEP was considerably stronger in more severe ARDS patients than in less severe individuals. A previous meta-analysis showed that a high PEEP reduced mortality in patients with ARDS but not ALI [8] However, in the present study, there was no difference of hospital mortality between groups of ARDS patients with a baseline PaO_2_/FiO_2_ less than 150 mmHg.

The purpose of PEEP is to recruit collapsed alveoli in a gravity-dependent lung but not to induce the alveoli to overdistend in the non-dependent lung. Patients with ARDS exhibit significant dependent atelectasis due to increased lung weight resulting from interstitial and alveolar edema. Both higher PEEP and recruitment manoeuvres can reduce atelectasis and increase end-expiratory lung volume. Physically, we need higher pressure to open alveoli and only need a relative lower PEEP to keep the lung open. Therefore, it was noted that higher PEEP should follow the recruitment manoeuvres. Goligher et al. showed that combine with recruitment manoeuvres and higher PEEP could reduce mortality of patients with ARDS [18]. The results were also demonstrated in the study by Constantin et al. [21]. However, the study by Goligher et al. [10] indicated that the percentage of potentially respond to PEEP was extremely variable. Our results demonstrated the hypothesis that only patients who respond to increased PEEP by improved oxygenation benefitted more from higher PEEP. PEEP could have different effects on clinical outcomes based on the nature of the clinical response to PEEP itself. In ARDS patients with clinically positive oxygenation response to PEEP, the main effect of a high PEEP might be recruiting the collapsed lung, which may reduce ventilation-induced lung injury (VILI). In contrast, a high PEEP might result in alveolar overdistension, which induces lung injury in ARDS patients without positive oxygenation response to PEEP. These reasons could help to explain why our subgroup analysis showed that high PEEP reduced mortality in ARDS patients with clinically positive oxygenation response to PEEP.

The severity of ARDS can be used to titrate PEEP. There are many methods for PEEP titration, such as oxygenation, stress index and transpulmonary pressure. Chiumello et al. found that oxygenation-guided PEEP provided PEEP levels related to oxygenation response to PEEP in the lung that progressively increased from mild to moderate and severe ARDS [19]. In assessing the oxygenation response to PEEP at 5 cmH_2_O PEEP based on the Berlin definition [6, 22], a simple Friday-night ventilation strategy can be used in patients with ARDS [23]. Therefore, while we only included studies in which the PEEP level was lower than 10 cmH_2_O in the low PEEP group, decreased ICU mortality was found in the high PEEP group.

There are many methods that can be used with ARDS patients at present. To date, prolonged sessions of prone positioning has not only been shown to be efficient at significantly increasing oxygenation and decreasing driving pressure in severe ARDS in the APRONET study [24], but the PROSEVA trial also showed that prone positioning was associated with improved patient survival [25]. However, the LUNG SAFE study showed that the rate of prone positioning was low because of selection bias or the clinician’s perception [26]. On the other hand, neuromuscular blockers could be used in severe ARDS, but they are controversial. Many previous studies found that the mortality of ARDS treated with neuromuscular blockers was not decreased. However, neuromuscular blockers could exert beneficial effects in patients with moderate ARDS, at least in part, by limiting expiratory efforts as shown in Guervilly’s study [27]. In our study, there were 3 RCTs that included prone position or neuromuscular blockers. Unfortunately, due to the lack of prognostic data, statistical analysis was not possible.

We did not find a significant difference in clinically objectified barotrauma between high PEEP and low PEEP groups. Lung-protective strategies were used in all of the included studies, which decreased the incidence of clinically objectified barotrauma [28, 29]. In addition, most patients in these studies had moderate or severe ARDS, which indicates that more lung area was responsive to increased PEEP. However, in ARDS patients without positive oxygenation response to PEEP, high PEEP increased the incidence of clinically objectified barotrauma. It was shown in a recent study that an exceedingly high pressure was used to recruit the lung, especially in ARDS patients without positive oxygenation response to PEEP, and mortality may increase [8].

Some limitations of our analysis should be noted. First, we were unable to obtain patient-level data despite asking for these data from the authors of the included studies. The lung oxygenation response to PEEP was evaluated based on the mean PaO_2_/FiO_2_ values reported in each study but not the individual patient data. That finding means most of the patients in the study with positive oxygenation response to increased PEEP were in their respective groups.

Second, the definition of positive oxygenation response to PEEP based on Goligher’s study is not highly precise [10]. We also did not assess other factors that may affect the oxygenation response to PEEP. These factors may have affected the accuracy of our results. In this meta-analysis, we only included the data from the included studies. In addition, we did not used the data on SpO_2_, PaCO_2_ and respiratory compliance at the two different PEEP levels. We only used the PaO_2_/FiO_2_ data at higher and lower PEEP levels. Therefore, we defined the groups as positive oxygenation response to PEEP if the mean PaO_2_/FiO_2_ was higher in the high PEEP group than at baseline. Based on this definition, we found that patients with clinically positive oxygenation response to PEEP benefitted from high PEEP.

Third, the methods of PEEP titration were different between the included studies, which may have induced bias in our study. Oxygenation response to PEEP should be considered during PEEP titration in patients with ARDS. However, no clinical trial has shown that oxygenation response to PEEP using assessment–guided PEEP titration could improve the outcomes in patients with ARDS, which indicates further studies are needed.

## Conclusion

In this study, high PEEP did not improve outcomes in patients with ARDS as a whole. However, subgroup analysis showed that high PEEP decreased hospital and ICU mortality in ARDS patients with a clinically objective positive oxygenation response to PEEP.

## Additional files

Additional file 1: **Table S1.** Description of Patients, Interventions, Comparators, and Outcomes (PICO) targeted by the systematic review. (DOCX 42 kb)

Additional file 2: **Figure S1.** Funnel plot of high PEEP effect on hospital mortality in ARDS patients. (PNG 52 kb)

Additional file 3: **Figure S2.** funnel plot of high PEEP effect on hospital mortality while the PEEP level of patients in low PEEP group was different. (PNG 44 kb)

Additional file 4: **Figure S3.** Funnel plot of high PEEP effect on hospital mortality of moderate and severe ARDS patients between high and low PEEP groups. (PNG 57 kb)

Additional file 5: **Figure S4.** Funnel plot of high PEEP effect on 28-day mortality in ARDS patients. (PNG 53 kb)

Additional file 6: **Figure S5.** Funnel plot of high PEEP effect on 28-day mortality while the PEEP level of patients in low PEEP group was different. (PNG 42 kb)

Additional file 7: **Figure S6.** Funnel plot of high PEEP effect on ICU mortality in ARDS patients. (PNG 63 kb)

Additional file 8: **Figure S7.** Funnel plot of high PEEP effect on ICU mortality while the PEEP level of patients in low PEEP group was different. (PNG 53 kb)

Additional file 9: **Figure S8.** Funnel plot of high PEEP effect on clinically objectified barotrauma in ARDS patients. (PNG 46 kb)

## Abbreviations

ALI: Acute lung injury
ARDS: Acute respiratory distress syndrome
ARF: Acute respiratory failure
ICU: Intensive care unit
PEEP: Positive end-expiratory pressure
RCT: Randomized controlled trials
VILI: Ventilation-induced lung injury
