# Supplementary material for: Higher PEEP improves outcomes in ARDS patients with clinically objective positive oxygenation response to PEEP: a systematic review and meta-analysis
Source: BMC Anesthesiol. 2018 Nov 17;18:172. doi: 10.1186/s12871-018-0631-4 (PMC6240288; doi:10.1186/s12871-018-0631-4)
Supplement: Supplementary file 1 — Table S1. Description of Patients, Interventions, Comparators, and Outcomes (PICO) targeted by the systematic review. (DOCX 42 kb) [file 12871_2018_631_MOESM1_ESM.docx]

Table S1 Description of Patients, Interventions, Comparators, and Outcomes (PICO) targeted by the systematic review.

| Questions | Description |
| --- | --- |
| Patient | Mechanically ventilated, adult patients with ARDS |
| Intervention | Higher PEEP |
| Comparators | Lower PEEP |
| Outcome | Primary outcome: mortality(28d, ICU,hospital)  Secondary outcome: barotrauma |
