# Supplementary figures and images for: Higher PEEP improves outcomes in ARDS patients with clinically objective positive oxygenation response to PEEP: a systematic review and meta-analysis
Source: BMC Anesthesiol. 2018 Nov 17;18:172. doi: 10.1186/s12871-018-0631-4 (PMC6240288; doi:10.1186/s12871-018-0631-4)

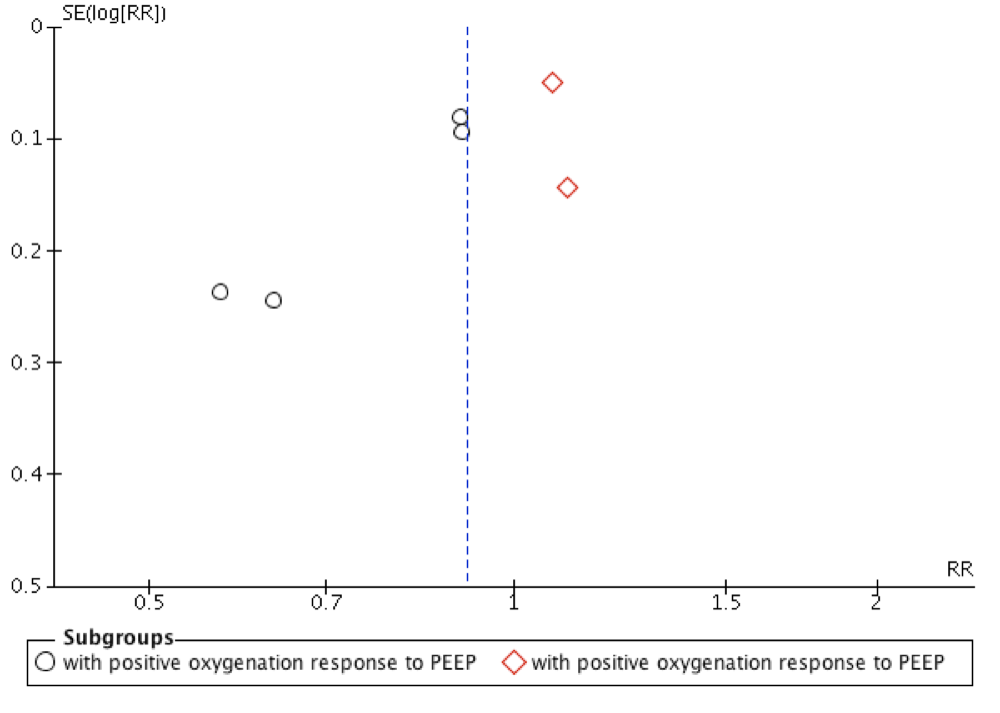

Supplement: Supplementary file 2 — Figure S1. Funnel plot of high PEEP effect on hospital mortality in ARDS patients. (PNG 52 kb) [file 12871_2018_631_MOESM2_ESM.png]

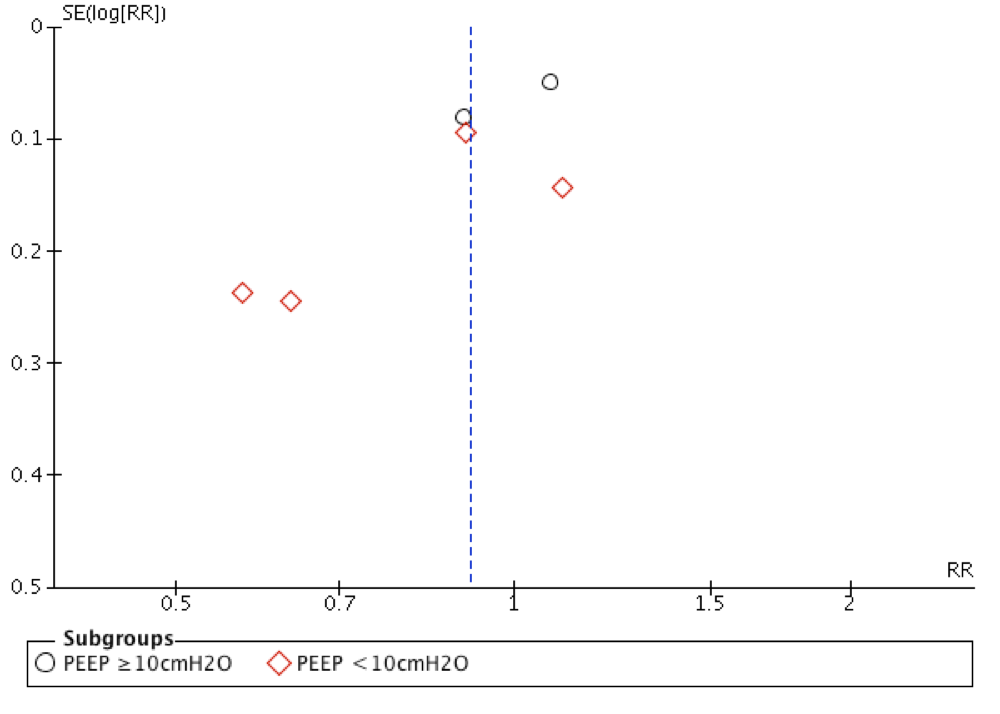

Supplement: Supplementary file 3 — Figure S2. funnel plot of high PEEP effect on hospital mortality while the PEEP level of patients in low PEEP group was different. (PNG 44 kb) [file 12871_2018_631_MOESM3_ESM.png]

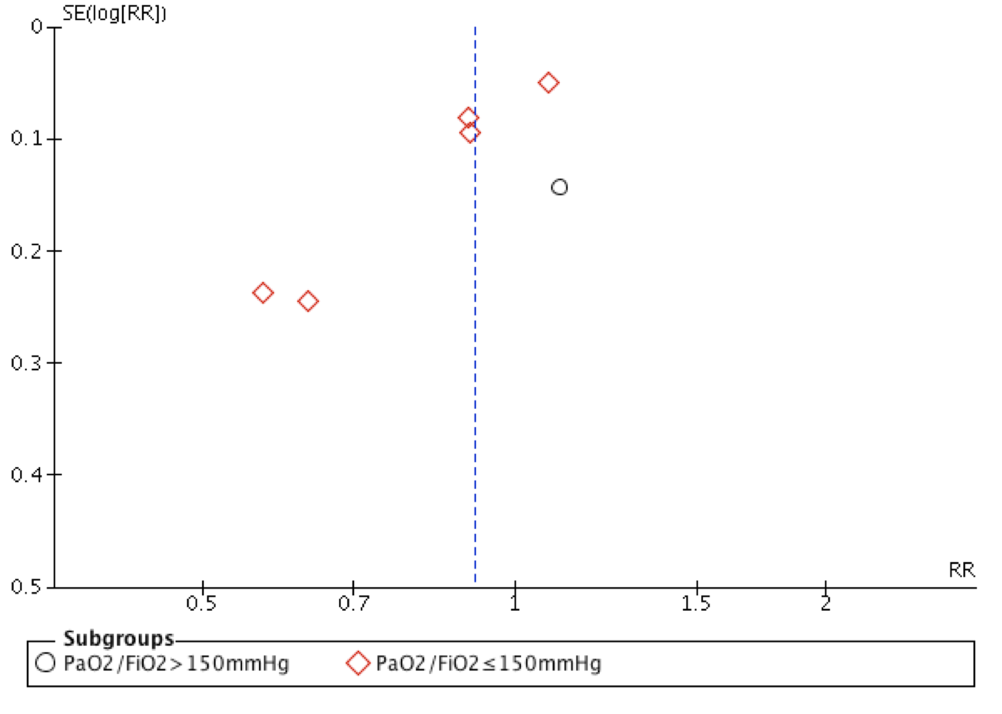

Supplement: Supplementary file 4 — Figure S3. Funnel plot of high PEEP effect on hospital mortality of moderate and severe ARDS patients between high and low PEEP groups. (PNG 57 kb) [file 12871_2018_631_MOESM4_ESM.png]

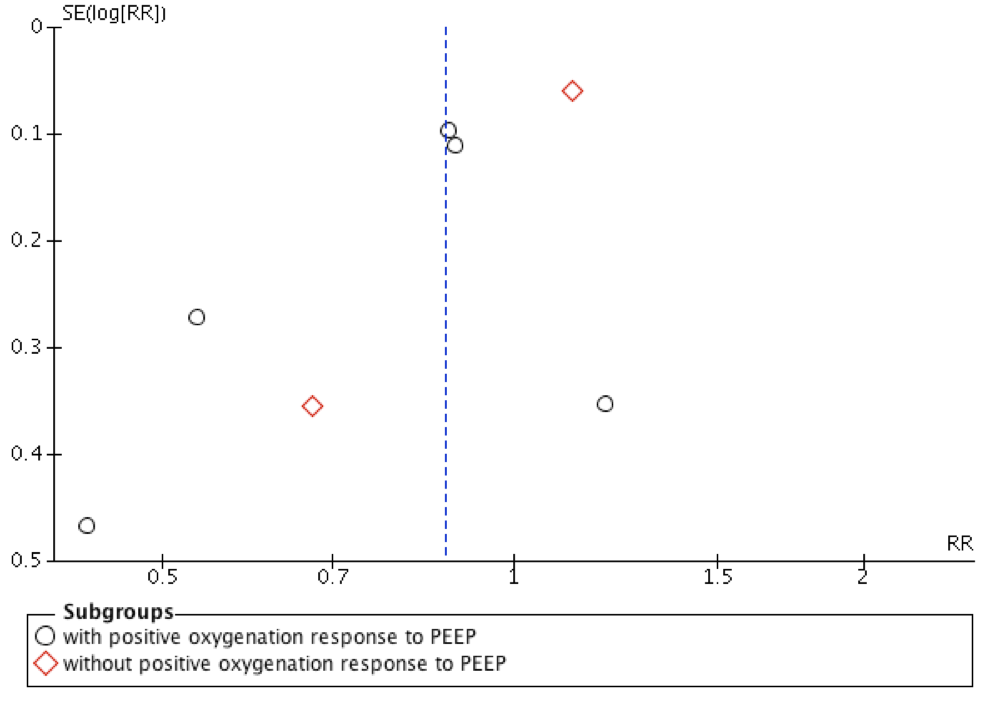

Supplement: Supplementary file 5 — Figure S4. Funnel plot of high PEEP effect on 28-day mortality in ARDS patients. (PNG 53 kb) [file 12871_2018_631_MOESM5_ESM.png]

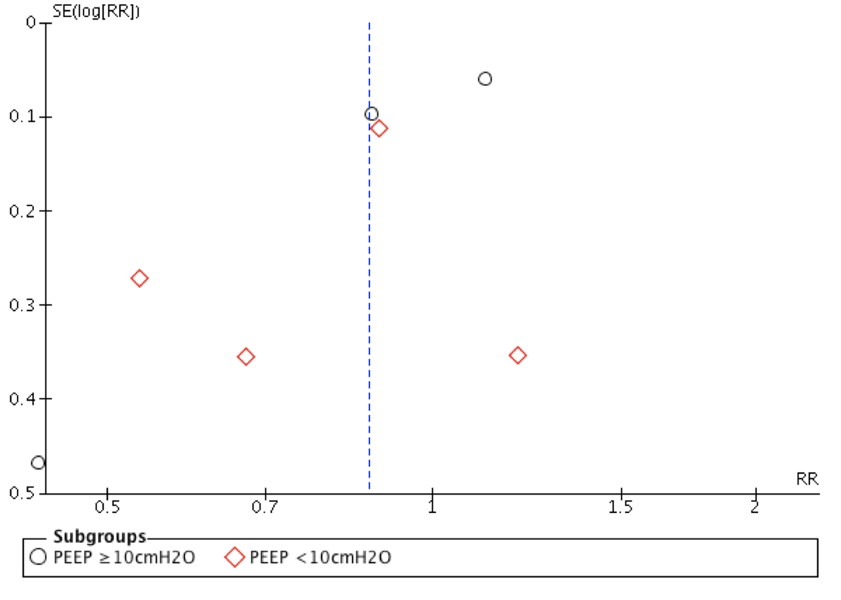

Supplement: Supplementary file 6 — Figure S5. Funnel plot of high PEEP effect on 28-day mortality while the PEEP level of patients in low PEEP group was different. (PNG 42 kb) [file 12871_2018_631_MOESM6_ESM.png]

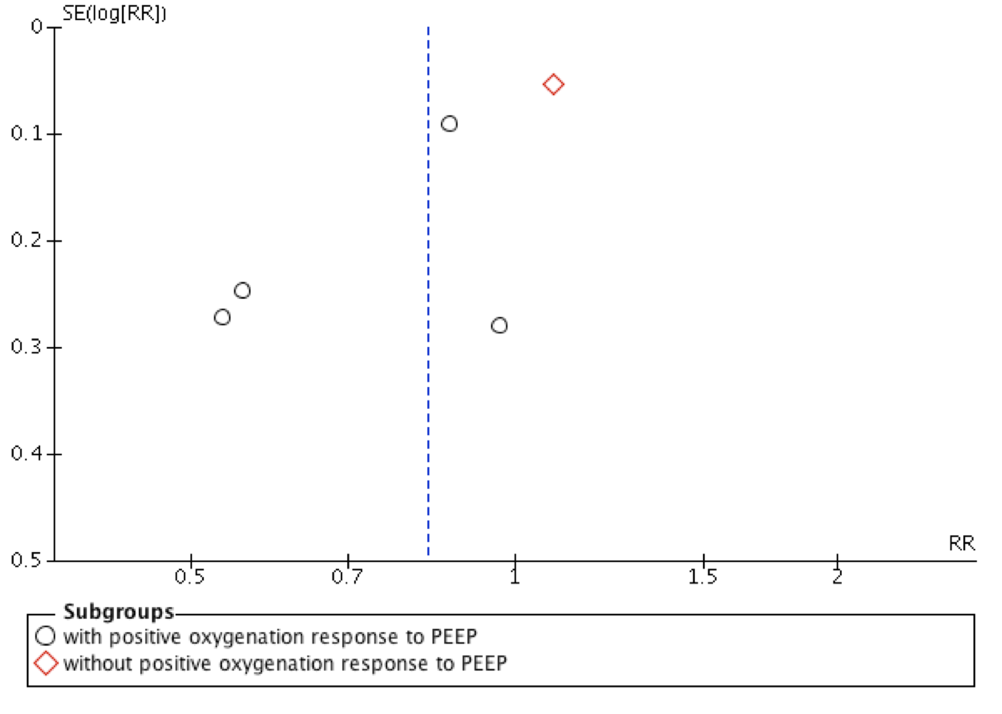

Supplement: Supplementary file 7 — Figure S6. Funnel plot of high PEEP effect on ICU mortality in ARDS patients. (PNG 63 kb) [file 12871_2018_631_MOESM7_ESM.png]

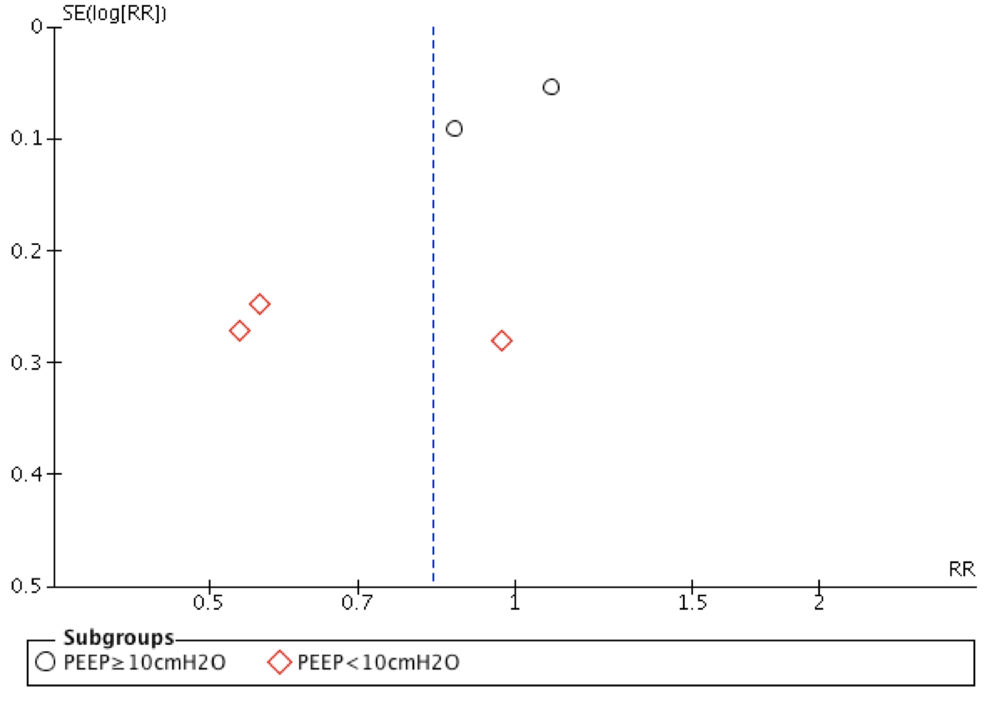

Supplement: Supplementary file 8 — Figure S7. Funnel plot of high PEEP effect on ICU mortality while the PEEP level of patients in low PEEP group was different. (PNG 53 kb) [file 12871_2018_631_MOESM8_ESM.png]

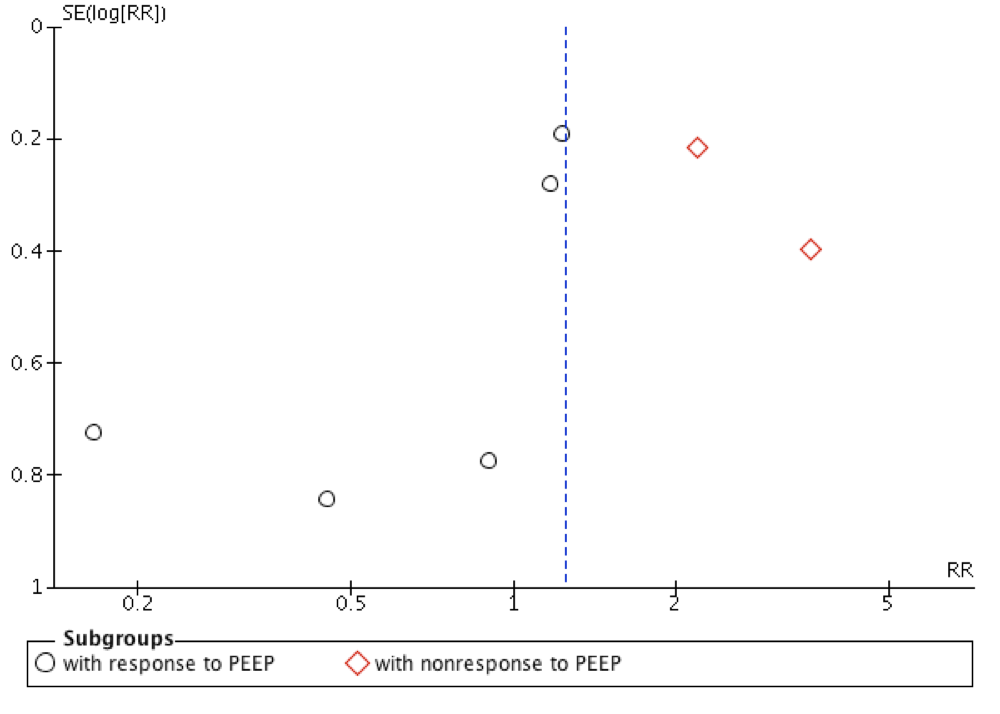

Supplement: Supplementary file 9 — Figure S8. Funnel plot of high PEEP effect on clinically objectified barotrauma in ARDS patients. (PNG 46 kb) [file 12871_2018_631_MOESM9_ESM.png]
